# Hydroxyapatite Coating of Titanium Implants Using Hydroprocessing and Evaluation of Their Osteoconductivity

**DOI:** 10.1155/2012/730693

**Published:** 2012-02-09

**Authors:** Kensuke Kuroda, Masazumi Okido

**Affiliations:** Department of Materials Science and Engineering, Graduate School of Engineering, Nagoya University, Nagoya 464-8603, Japan

## Abstract

Many techniques for the surface modification of titanium and its alloys have been proposed from the viewpoint of improving bioactivity. This paper contains an overview of surface treatment methods, including coating with hydroxyapatite (HAp), an osteoconductive compound. There are two types of coating methods: pyroprocessing and hydroprocessing. In this paper, hydroprocessing for coating on the titanium substrate with HAp, carbonate apatite (CO_3_–Ap), a CO_3_–Ap/CaCO_3_ composite, HAp/collagen, and a HAp/gelatin composite is outlined. Moreover, evaluation by implantation of surface-modified samples in rat tibiae is described.

## 1. Introduction

Titanium (Ti) and its alloys are used as artificial joints and teeth roots in orthopedic and dental settings because they have the advantage that their mechanical properties are closer to those of bone than are those of stainless steel or cobalt-chromium alloys. However, the difference in mechanical properties between Ti and natural bone leads to negative effects, such as stress shielding. To mitigate these effects, many new Ti alloys have been developed for hard tissue implants, with a focus on controlling the alloy element and its content, phase, and other characteristics.

When implants do not undergo surface modification to enhance the osteoconductivity, it takes a relatively long time to fix the metallic implant to bone such that it is stable. There are many approaches for improving the osteoconductivity of Ti and its alloys. These approaches can be classified into the following two techniques: (1) bioactive compounds that accelerate bone formation are coated on metallic implants and (2) a rough surface at the macrolevel is formed on the metallic implants, and the ingrowth of bone results in anchorage of the implants. These techniques have achieved a certain level of success, and the surface-modified implants have been used clinically. However, there are still weaknesses with the coating that need resolution, as well as unclear points regarding the effect of the surface properties on the osteoconductivity. Since hydroprocessing can be used to prepare the coating on complex-shaped substrates with complex topography, which many implants have, we focus on the use of hydroprocessing in many techniques for coating the bioactive compound, especially hydroxyapatite (HAp), and expound on the characteristics of the techniques and issues. Moreover, we describe in detail the evaluation of the osteoconductivity of implants coated with HAp, using *in vivo* testing in rat tibiae.

## 2. HAp Coating

HAp (Ca_10_(PO_4_)_6_(OH)_2_), which is the main inorganic component in the mammal bone or tooth [1], has attracted attention as a surface-coating compound because of its high osteoconductivity. Many pyroprocessing methods of forming HAp and other calcium phosphate coatings on metallic substrates have been reported (e.g., plasma spraying [2, 3], sol-gel method [4, 5], electron beam sputtering method [6], and ion beam sputtering method [7]). However, all have weak points in relation to coating with HAp on complex-shaped implants. Plasma spraying remains the most commonly used technique for HAp coating on a Ti or Ti alloy substrate in the fabrication of artificial joint replacements [1] and in endosseous dental implants [2]. On the other hand, many hydrocoating techniques (e.g., cathodic electrolysis method [8–10], electrophoretic method [11, 12], and thermal substrate method [13–18]) have been proposed as approaches to forming thin-film coatings on metallic substrates. The cathodic electrolysis and thermal substrate methods are single-step coating techniques in an aqueous solution, and they coat the HAp directly from the solution. The electrophoretic method is omitted from this paper because it uses HAp formed by other methods in advance, despite the hydroprocessing. Therefore, in this paper, we describe the cathodic electrolysis and thermal substrate methods.

### 2.1. Theory of HAp Coating Using Hydroprocessing

It is known that the solubility of HAp in an aqueous solution decreases with increasing temperature and that the relationship between the HAp solubility product, *K*_SP_/(mol dm^−3^)^9^, and the temperature, *T*/*K*, is given by [19]

(1) $${\log\;K}_{\text{SP}}=-\frac{8219.41}{T}-1.6657-0.098215\;T.$$

Therefore, heating an aqueous solution containing Ca^2+^ and PO_4_^3−^ ions results in the precipitation of calcium phosphates, such as HAp, in the solution.

**Figure 1:**
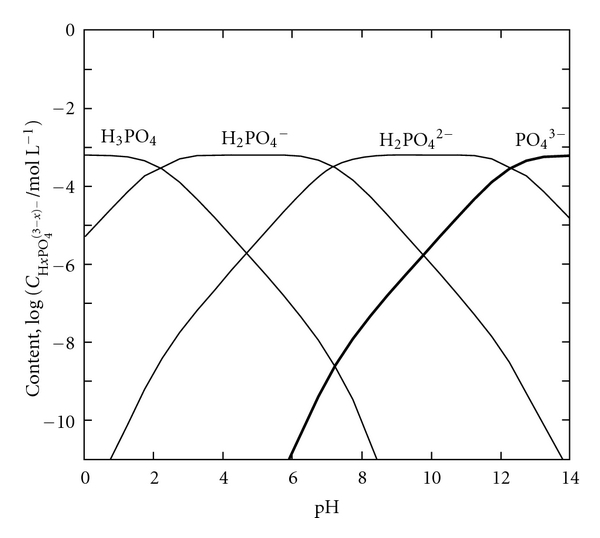
Logarithmic concentration diagram for orthophosphoric acid.

The ionic product of HAp, *K*_IP_/(mol L^−1^)^9^, is expressed as follows:

(2) $$K_{\text{IP}}=\;{\left[\text{C}{\text{a}}^{2+}\right]}^{5}{\left[{{\text{PO}}_{4}}^{3-}\right]}^{3}\left[{\text{OH}}^{-}\right],$$

where [*X*] indicates the molar concentration (mol L^−1^) of ionic species *X*. The increase in [Ca^2+^] or [PO_4_^3−^] content or pH value in the solution initiates the precipitation of HAp because *K*_IP_ achieves *K*_SP_. Moreover, [PO_4_^3−^] increases with increasing pH value (Figure 1). Therefore, the increase in pH directly accelerates the precipitation of HAp, which indirectly increases the [PO_4_^3−^] content.

**Figure 2:**
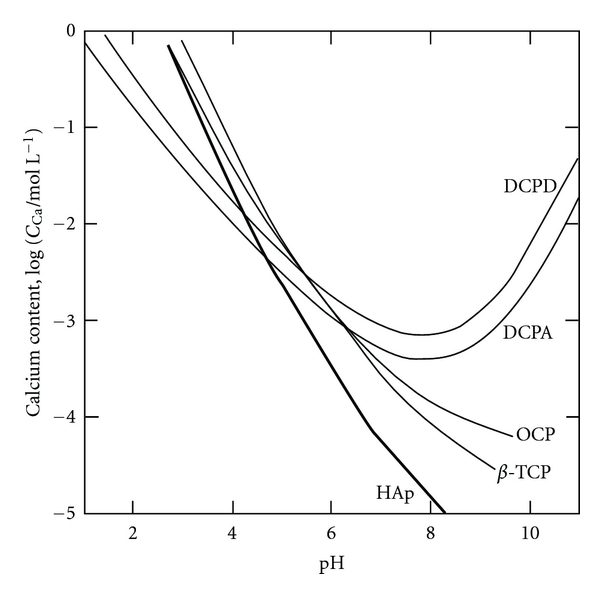
Solubility curves of calcium orthophosphoric compounds at 37°C, depending on pH in aqueous solution. HAp: hydroxyapatite (Ca_10_(PO_4_)_6_(OH)_2_), TCP: calcium phosphate (Ca_3_(PO_4_)_2_), OCP: octacalcium phosphate (Ca_8_H_2_(PO_4_)_6_ 5H_2_O), DCPA: dicalcium phosphate anhydrous (CaHPO_4_), DCPD: dicalcium phosphate dihydrate (CaHPO_4_ 2H_2_O).

Figure 2 shows the solubility curves of various compounds on calcium orthophosphate [19]; as shown, there are many compounds other than HAp. This figure indicates that CaHPO_4_ (DCPA) is the most stable compound at pH < 5, with HAp the most stable at pH > 5. Therefore, HAp can be easily obtained in a solution where pH > 5 and where the ion content and temperature are controlled. However, HAp cannot precipitate in a solution of pH < 5, and hydroprocessing using the precipitation phenomenon in the aqueous solution cannot give *β*-Ca_3_(PO_4_)_2_ (*β*-TCP), a bioactive compound.

### 2.2. Thermal Substrate Method in Aqueous Solution [13]

**Figure 3:**
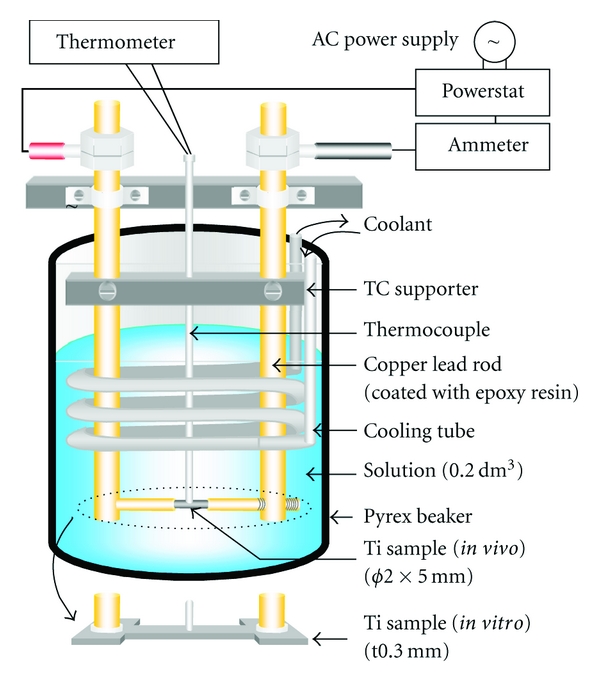
Experimental apparatus for HAp coating.

This process involves passing an alternating current through a metallic sample immersed in an aqueous solution. The immersed metallic sample heats up to more than 100°C by Joule heating, even though the hydroprocessing occurs at atmospheric pressure (Figure 3). Therefore, this method can produce the special reaction conditions (>100°C in an aqueous solution) by not using the pressure vessel.

**Figure 4:**
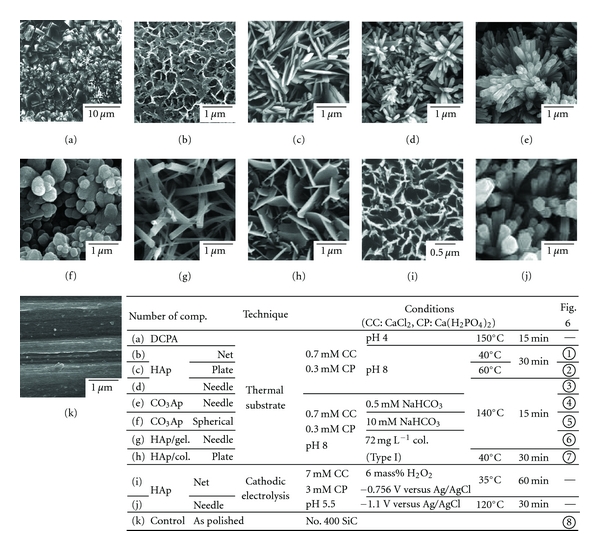
SEM photographs of the surface of the samples treated by various methods.

**Figure 5:**
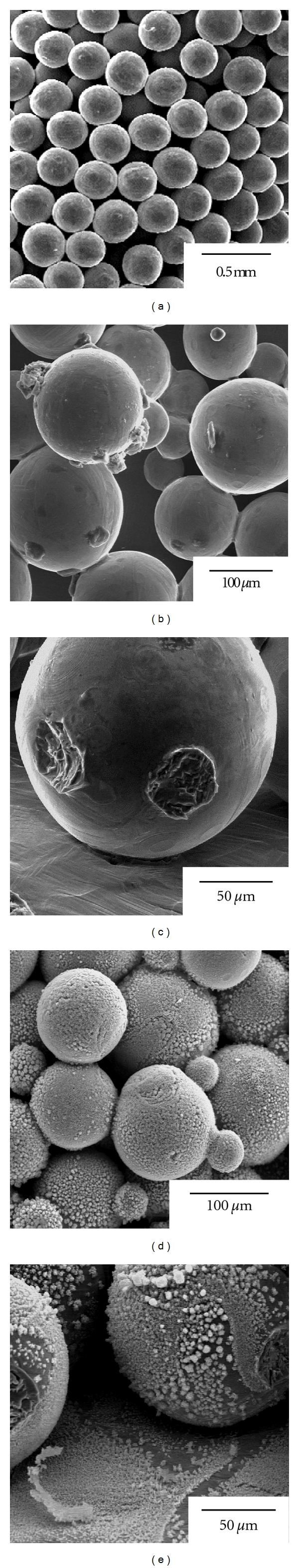
Surface and/or cross-sectional views of the samples with surface roughness. (a)–(c) bead-sintered porous samples (as sintered), (d)-(e) bead-sintered porous samples coated with HAp (thermal substrate method, 0.7 mM CaCl_2_ + 0.3 mM Ca(H_2_PO_4_)_2_, pH 7, 100°C, 15 min.).

When the thermal substrate method in an aqueous solution is used, the fact that the solubility of HAp decreases with increasing temperature means that HAp precipitation occurs only on the substrate. It is important to coat HAp while controlling the concentration of the solute and the pH value of the solution and temperature, because they affect the degree of supersaturation of HAp in the solution (Equations (1) and (2)). Figures 4(a)–4(d) show the change in the surface morphology of the samples coated with HAp under controlled pH and temperature, whose factors determined the degree of the supersaturation with respect to HAp [14, 15, 17, 18]. The precipitate at pH = 4.0 (Figure 4(a)) appeared to pile up like bricks and was identified as DCPA, which is a stable compound. On the other hand, in the solution at pH = 8.0, the precipitate was composed of HAp (Figures 4(b)–4(d)). From the EDX analysis, the molar ratio of calcium to phosphorous (Ca/P) of HAp was 1.41–1.43. This shows the coated HAp was calcium deficient. The pH dependence of the solubility of the calcium phosphate compounds explains why the precipitate changed with increasing pH of the solution, that is, the solubility curves of DCPA and HAp cross at approximately pH = 5 for various compounds of calcium phosphate [19]. The surface morphology of the precipitated HAp strongly depends on coating temperature: low temperature (40°C) gave net-like HAp (Figure 4(b)); high temperature (140°C) gave needle-like HAp (Figure 4(d)); mid temperature (60°C) gave plate-like HAp (Figure 4(c)). That is, by using hydroprocessing, we can control the crystalline form, which could not have been achieved using traditional methods.  Figure 5 shows the scanning electron microscopy (SEM) photographs of the HAp-coated samples on porous Ti alloy surfaces formed by sintering Ti6Al4V particles (ca. 100 *μ*m in diameter) on cpTi substrates [16]. Heating at 100°C for 15 min. in a pH = 7 solution led to HAp precipitation over the entire surface of the Ti6Al4V sintered particles (on both front and back faces) and on the base cpTi substrate of the experimental samples. In particular, it was found that HAp precipitate was also detected at the sinter neck regions of adjacent particles and on the base substrate, while the original open-pored geometry was maintained. Therefore, this method can be used to apply the HAp coating to a substrate with complex topography.

Biological apatite in natural bone does not appear in the form of pure Hap, and it contains a considerable amount of carbonate ions [20] (about 7.4 mass% with respect to total bone and 11.4 mass% with respect to the inorganic component in natural bone [21]). Carbonate apatite (CO_3_–Ap), which replaces PO_4_^3−^ and/or OH^−^ ions with CO_3_^2−^ ions, is similar to the inorganic component of bone, and it seems to be a more promising bioactive material than stoichiometric HAp, because CO_3_–Ap has greater solubility than pure HAp [20]. In addition, it has been reported that CaCO_3_ displays bioactivity, such as cell compatibility and hard tissue compatibility [22, 23]; that is, CO_3_^2−^ is expected to influence biological reactivity and osteoconductive properties. It is also well known that the solubility of CaCO_3_ in an aqueous solution decreases with increasing temperature [24]. In the solution, when CO_3_^2−^ ions are added, CO_3_–Ap or CO_3_–Ap/CaCO_3_ composite films are easily obtained on a substrate. Typical SEM photographs of the surface of the samples are shown in Figures 4(e)-4(f), coated in a solution of pH = 8 with <0.5 mM NaHCO_3_ added at 140°C for a period of 15 min., and after the steam autoclaving treatment (5 mass% CO_3_ in this film) [25, 26]. The precipitates coated from the solution with >0.5 mM NaHCO_3_ added contained CO_3_–Ap and CaCO_3_ at all temperatures, and the X-ray diffraction spectra showed a mixture of calcite, vaterite, and aragonite. The crystalline form of CO_3_–Ap was changed, depending on the added NaHCO_3_ content, as well as coating temperature. In particular, adding a significant amount of NaHCO_3_ (>5 mM) brought about sphere-like CO_3_–Ap (Figure 4(f)) in the 140°C coating. In CO_3_–Ap films, FT-IR analysis revealed that CO_3_^2−^ was substituted for PO_4_^3−^ (Type B CO_3_–Ap) in advance, which was similar to biological apatite [27], and adding more CO_3_^2−^ to the solution gave the substitution for OH^−^ (type A). Therefore, in the samples with <0.5 mM NaHCO_3_ added, type B CO_3_–Ap was obtained, and in the samples with >5 mM NaHCO_3_ added (i.e., having the binary phase of CO_3_–Ap/CaCO_3_), type AB CO_3_–Ap was formed.

Natural bone contains CO_3_–Ap and a considerable amount of organic components, such as collagen (about 23 mass% [21]). It is known that the hybrid organic-inorganic structure initiates pliable bone. Some researchers have reported the preparation of nanocomposites of HAp/collagen and HAp/gelatin [28–30], as natural bone is considered a nanocomposite of mineral and proteins. Moreover, immobilization of collagen on implants displays a tighter fixation with the surrounding tissue, since the collagen behaves as an adhesive protein with cells because of the amino groups in the collagen molecules [31, 32]. From the viewpoint of osteoconductivity, we expected that preparing the HAp/collagen composite coating would be a more promising approach than using an individual coating of either CO_3_–Ap or HAp. In the solution to which acid-soluble collagen is added, HAp/collagen or HAp/gelatin composite films are easily obtained on a substrate, depending on coating temperature. In general, as mammalian collagen rapidly denatures to gelatin at >45°C, HAp/collagen composite can be obtained at <40°C and HAp/gelatin composite at >50°C. Figures 4(g)-4(h) show the surface of the samples coated in a solution of pH = 8 with 72 mg L^−1^ collagen, derived from calf, at 140°C and 40°C (10–15 mass% collagen or gelatin in the film) [33]. The surface morphologies of HAp/collagen and HAp/gelatin significantly depend on the coating temperature and are not affected by whether the composite film contains collagen or gelatin. That is, collagen and gelatin have only a small effect on the HAp crystal growth of the adsorption onto HAp. Hydroprocessing can be used to form HAp/collagen and HAp/gelatin composite films, which could not be formed using high-temperature processing, and the content of collagen and gelatin in the films can be controlled up to 60 mass%.

### 2.3. Cathodic Electrolysis in Aqueous Solution [8–10]

In the electrochemical technique, a redox reaction produces supersaturation of OH^−^ ions near the electrode in the aqueous solution containing Ca^2+^ and PO_4_^3−^ in the same manner as in the thermal substrate method. This local effect induces heterogeneous nucleation on the metal substrate serving as the electrode. The addition of hydrogen peroxide to the solution prevents H_2_ gas generation at the cathodic electrode and promotes nucleation and growth of the HAp coating. Adding H_2_O_2_ to electrolytes enhances the formation of OH^−^ ions at the solution-electrode interface at a lower cathodic potential, as described in the following reaction [10]:

(3) $${\text{H}}_{2}{\text{O}}_{2}+2{\text{e}}^{-}=2{\text{OH}}^{-}.$$

In this method, the surface morphology of the precipitated HAp greatly depends on coating temperature [34] in the same manner as in the thermal substrate method. The effect of temperature on the surface morphology of coated samples is shown in Figures 4(i)-4(j). The HAp crystals had a similar shape to those formed using the thermal substrate method, although the size of HAp crystals differed between the cathodic electrolysis and the thermal substrate methods. The molar ratio (Ca/P) of HAp was almost same as that using the thermal substrate method. The coatings at >100°C were conducted in the pressure vessel. When using the electrolysis solution, to which CO_3_^2−^ or collagen are added, CO_3_–Ap, HAp/collagen, or HAp/gelatin composite films are formed on a substrate, depending on coating temperature.

## 3. Evaluation of Osteoconductivity

The evaluation methods for the bioactivity of the implants are classified into *in vitro* and *in vivo *methods. In this review, the *in vivo* evaluation method is described. In *in vivo* evaluation, many types of animals at different ages were used in various studies, and different researchers used a different implanted part of the animals. Moreover, a unified quantification criterion has not yet been established, and the criteria used in various studies are not compatible with one another. Therefore, we use the bone-implant contact ratio, *R*_B-I_, as an osteoconductive index based on the observation of body tissue on the implants. Bone-implant contact was determined by linear measurement of direct bone contact with the implant surface. The sum of the length of the bone formation on the implant surface was measured and expressed as a percentage of the total implant length (bone-implant contact ratio) in the cancellous bone and the cortical bone parts [17, 18, 25, 26, 33]

(4) $$R_{\text{B-I}}\;\left(\%\right)=\frac{\text{sum}\;\text{of}\;\text{the}\;\text{length}\;\text{of}\;\text{the}\;\text{part}\;\text{of}\;\text{bone}\;\text{formation}\;\mathrm{on}\;\text{the}\;\text{implant}\;\text{surface}}{\text{total}\;\text{implant}\;\text{length}}\times 100.$$

**Figure 6:**
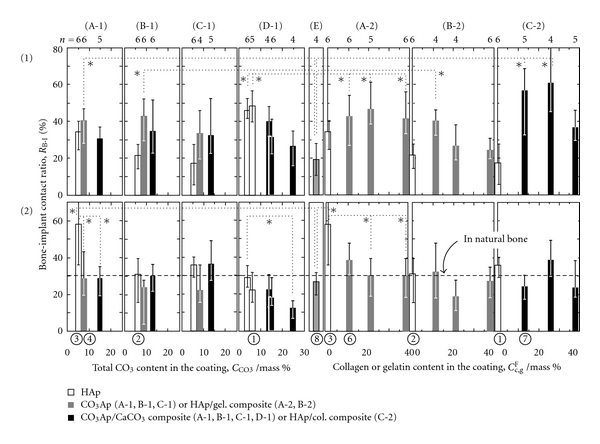
Bone-implant contact ratio, *R*_B-I_, for the various surface-coated samples (rats' tibia, 14 days). *P < 0.05 (1) cortical bone part, (2) cancellous bone part. (A) needle-like, (B) plate-like, (C) net-like, (D) spherical-like, (E) as-polished Ti.

Figure 6 shows the bone-implant contact ratios, *R*_B-I_, of the samples coated under the various conditions mentioned above and classified based on the following four surface morphologies: (A) needlelike, (B) platelike, (C) netlike, and (D) spherelike. The samples are then compared with the control implant ((E) noncoated Ti). In Figure 6, the samples are distinguished according to color based on whether or not the coating contained CaCO_3_ and collagen or gelatin (white: HAp; gray: CO_3_–Ap or HAp/gelatin; black: CO_3_–Ap/CaCO_3_ or HAp/collagen). The *R*_B-I_ value of HAp-coated samples (white bar) is the same as or higher than that of the as-polished one (E). In particular, *R*_B-I_ in the cancellous bone part is highest in the sample coated with the needle-like HAp (A-1). The influence of the different surface morphologies on *R*_B-I_ is apparent [17, 18]. A small amount of CO_3_ included in CO_3_–Ap does not influence osteoconductivity, and an increased amount of CO_3_ (>15 mass% CO_3_), including that in CO_3_–Ap/CaCO_3_, has a negative effect on (black bar in (A-1), (B-1), (C-1), and (D-1)) [25, 26]. The *R*_B-I_ value of HAp/gelatin-coated samples is the same as that of HAp (gray and white bars in (A-2) and (B-2)), and we did not find a positive effect of the addition to HAp on the osteoconductivity, or any negative effects within the limit of gelatin content used. In the HAp/collagen films (C-2), osteoconductivity was improved, and maximum *R*_B-I_ was obtained when the collagen content was the same as that in natural bone. The addition of too much collagen, exceeding that amount of collagen content in natural bone, inhibited the improvement of the osteoconductivity [33].

## 4. Conclusion

The inside of the human body is equivalent to a water environment at room temperature, since the water content in the body is about 60%. It is thought that hydroformed HAp has greater osteoconductivity than HAp synthesized using pyroprocessing, because synthesized HAp in the aqueous solution at neutral pH and room temperature is similar to that formed in the body. In addition, titanium dioxide, TiO_2_, which does not exist in the human body, is a remarkable compound with respect to its osteoconductivity. It is important to research and improve the osteoconductivity of substances such as HAp, TiO_2_, and CaTiO_3_. However, we need to pay attention to the properties of their compounds, such as surface roughness [35], crystallinity, and corrosivity, all of which influence osteoconductivity. Furthermore, the evaluation criterion for osteoconductivity has not been adequately established.

The development of implants with high functionality is an important problem that urgently needs to be solved, instead of merely making progress in medical technology. It is thought that nothing can compete with such implants in the progress and development of the individual technology. We hope that these important problems can be solved using the combination of the discovery of new bioactive compounds (organic and inorganic) and their coating techniques, alloy designs for the implants, and/or the growth of related surrounding techniques for them.
